# Supplementary material for: Comparative Analysis and Identification of Terpene Synthase Genes in Convallaria keiskei Leaf, Flower and Root Using RNA-Sequencing Profiling
Source: Plants (Basel). 2023 Jul 28;12(15):2797. doi: 10.3390/plants12152797 (PMC10421360; doi:10.3390/plants12152797)
Supplement: Supplementary file 1 [file plants-12-02797-s001.zip › supplementary files.pdf]

Article

Comparative analysis and identification of terpene synthase genes in *Convallaria keiskei* leaf, flower and root using RNA-sequencing profiling

Gurusamy Raman†§, Sivagami Jean Claude†, and SeonJoo Park*

Department of Life Sciences, Yeungnam University, Gyeongsan, Gyeongbuk 38541, South Korea.
* Correspondence: SeonJoo Park; sjpark01@ynu.ac.kr
§ Co-Correspondence: Gurusamy Raman; bioramg@yu.ac.kr

† Both authors contributed equally to this manuscript

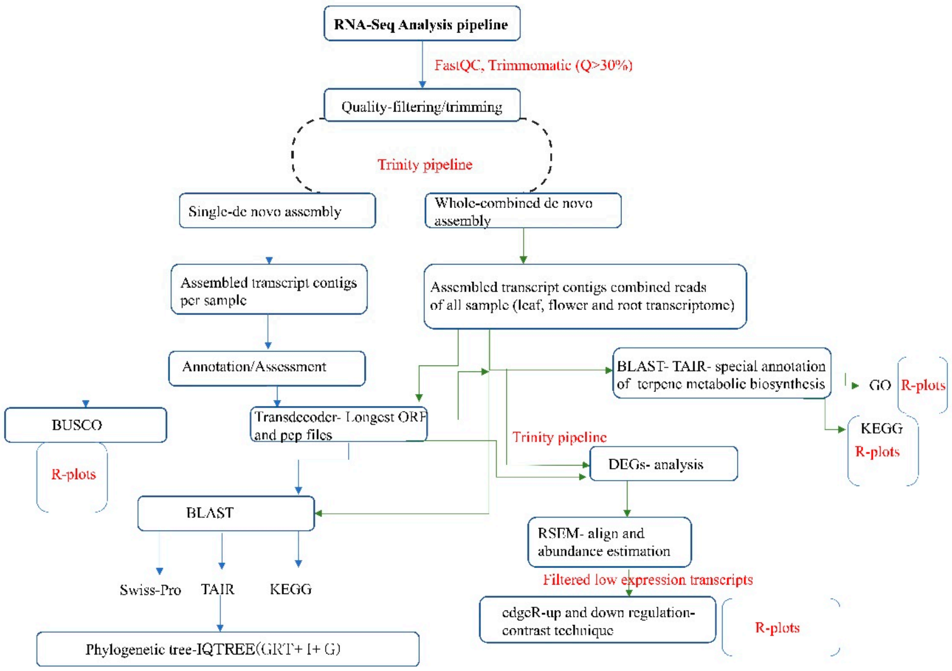

Supplementary Fig S1: Schematic diagram for a transcriptome assembly method

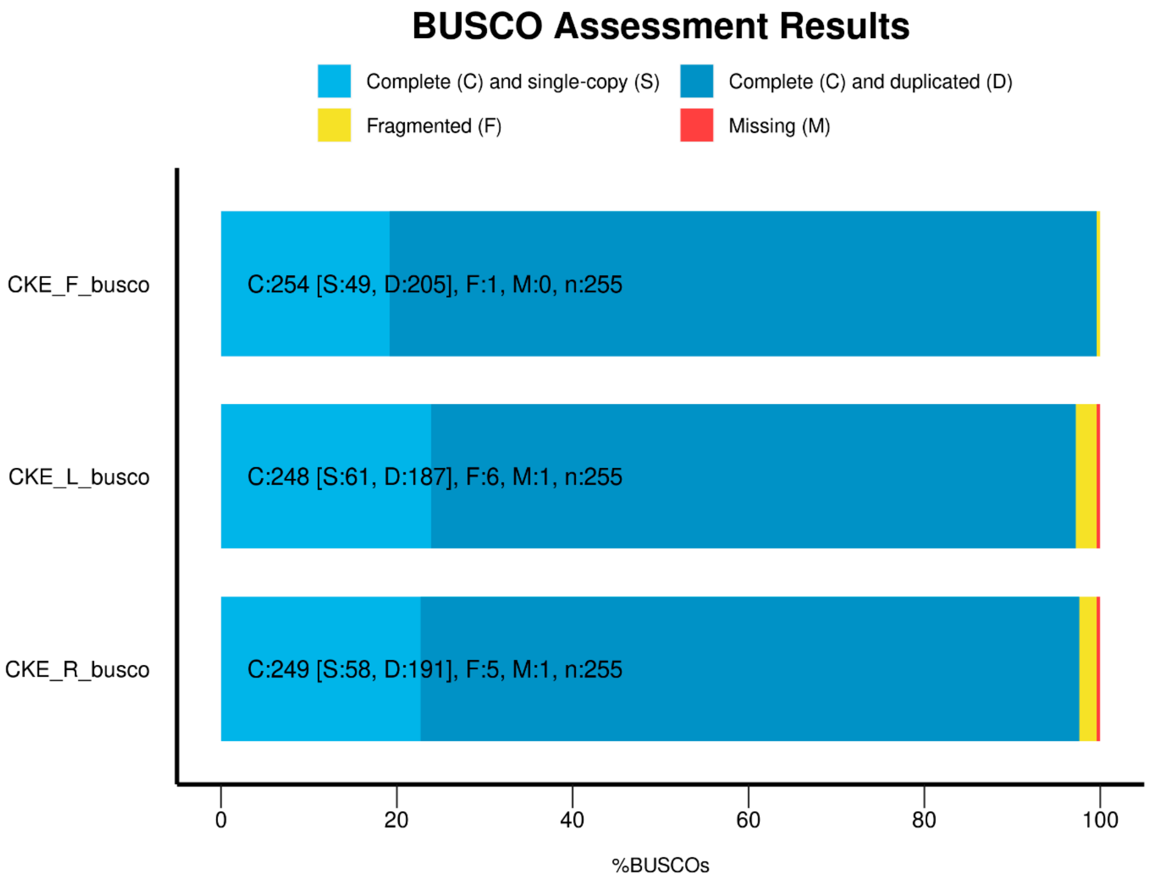

Supplementary Fig S2. BUSCO assessments of *Convallaria keiskei* transcriptome assembly

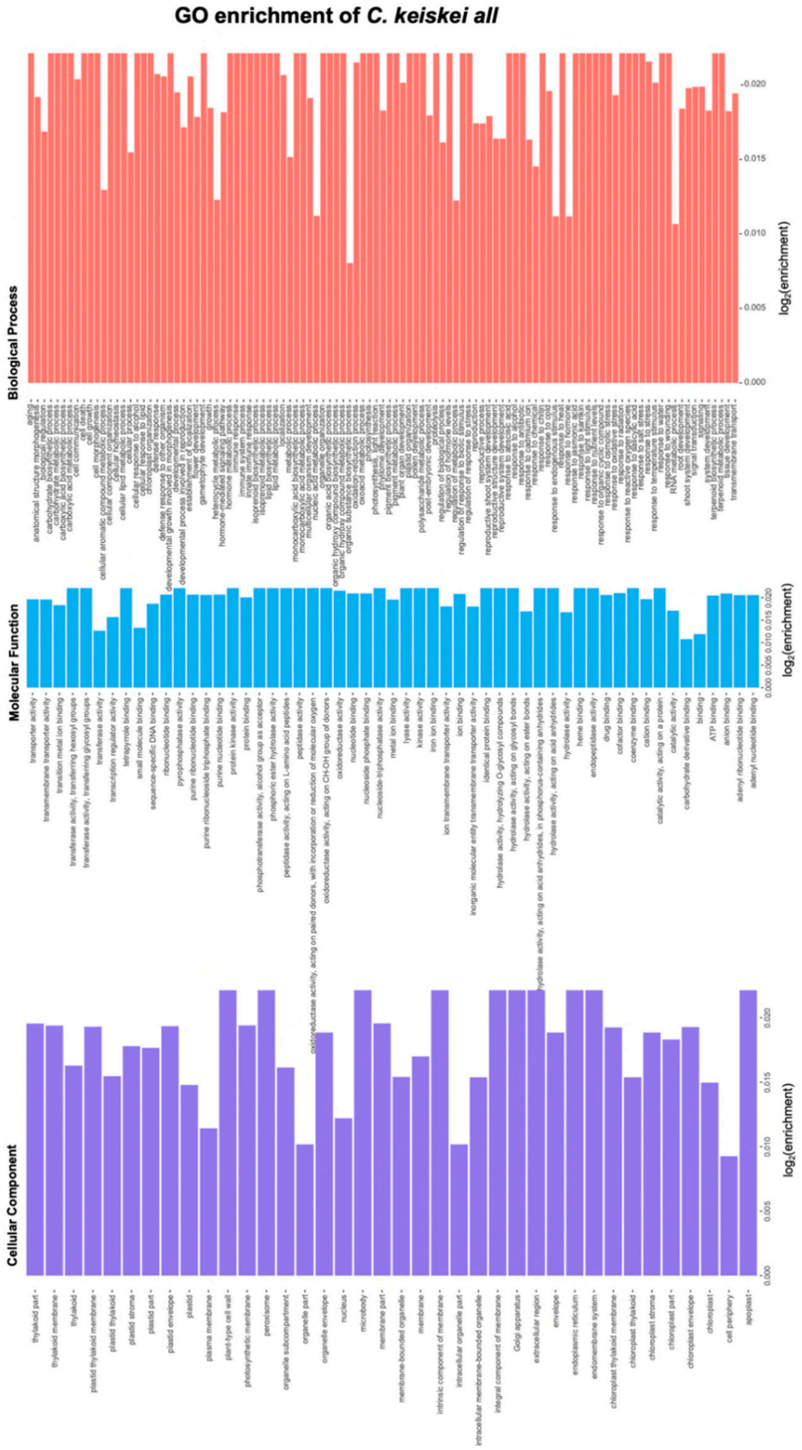

Supplementary Fig S3. Gene Ontology (GO) categories of combined transcriptome unigenes from the *C. keiskei* (TRAPID-enrichment analysis). (A) the distribution of biological process unigenes, (B) molecular function (C) cellular component in the *C. keiskei*

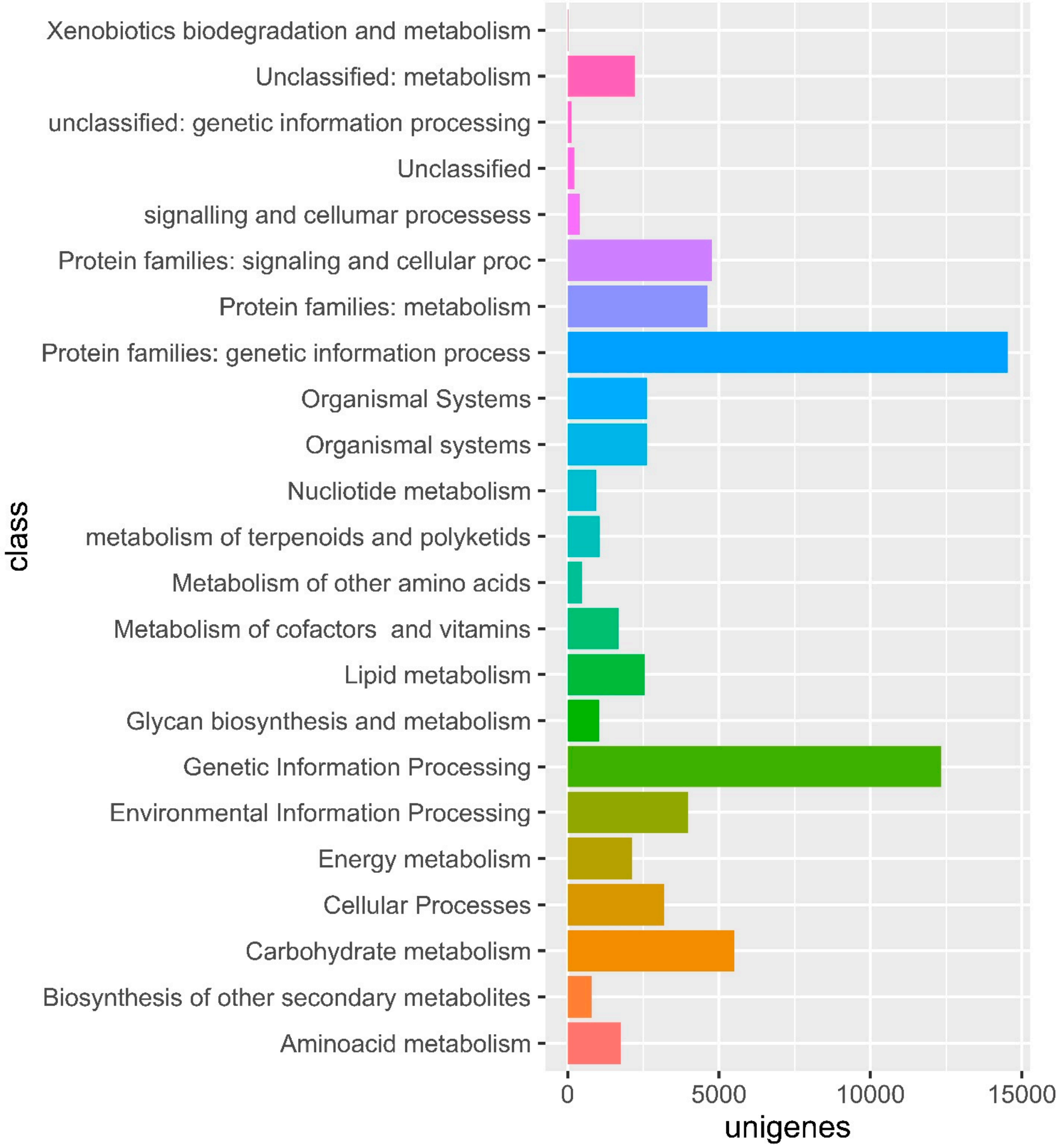

Supplementary Fig S4. The distribution of various classes of metabolic unigenes in the *C. keiskei* by KEGG analysis

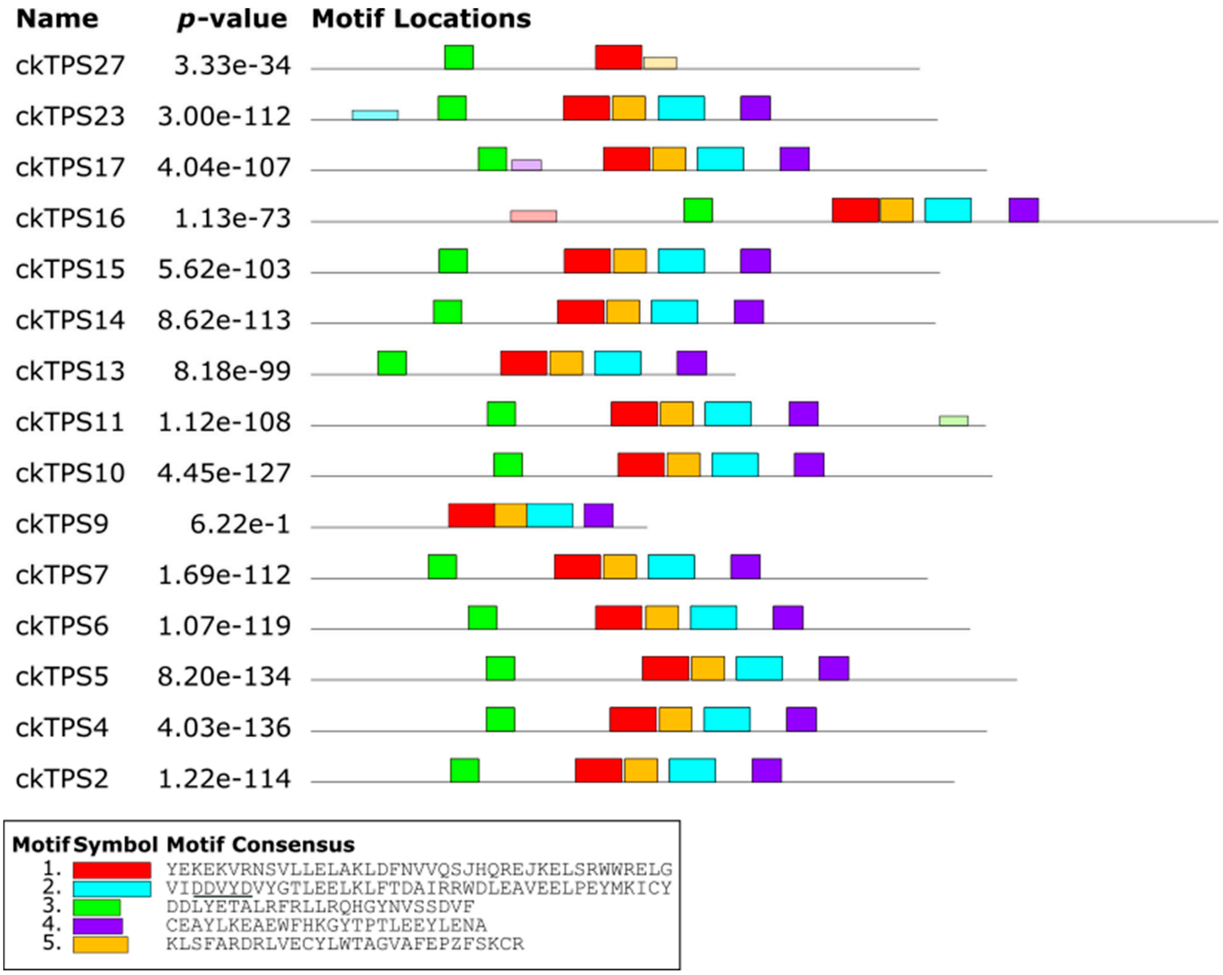

Supplementary Fig S5. Phylogenetic tree analysis and identification of linalool, nerolidol, and farnesol gene in *C. keiskei*. The green color rectangular box indicates the phylogenetic tree of all functional terpene synthase genes in the *C. keiskei*

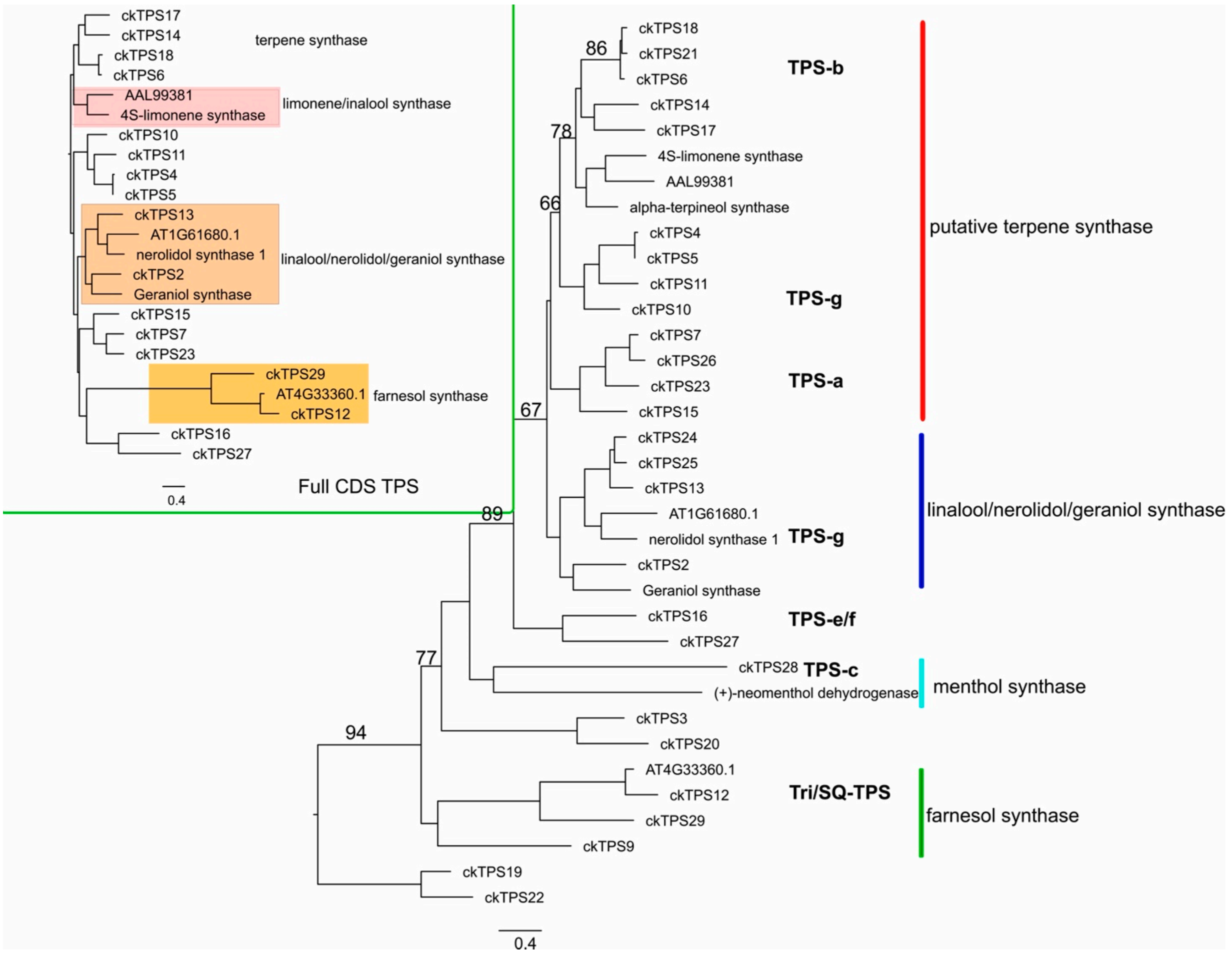

Supplementary Fig S6. MEME finding of the conserved domain (RRXXXD and DXXXD) of TPS in *Convallaria keiskei* transcripts

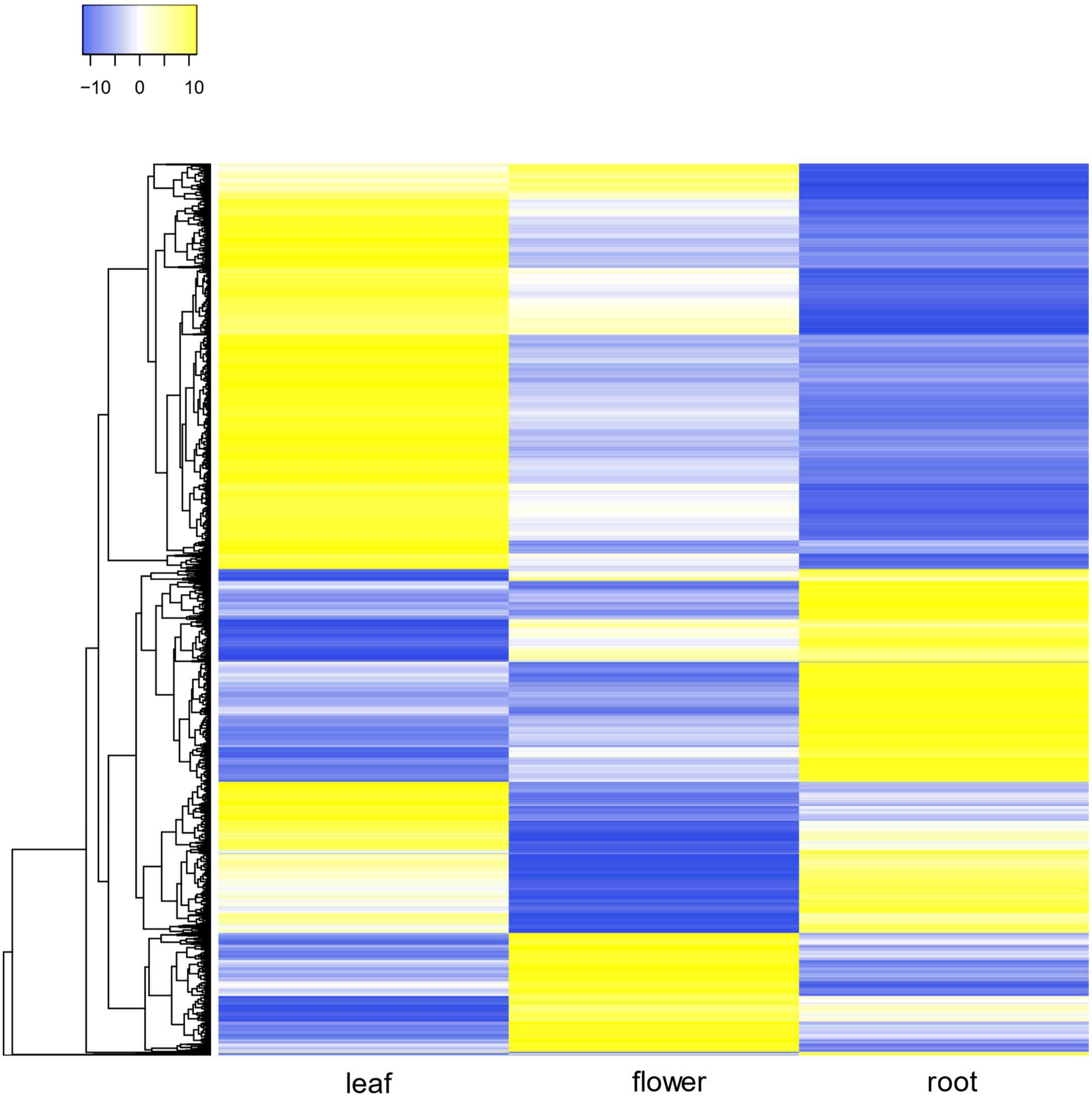

Supplementary Fig S7. Heatmap of differentially expressed 3,152 genes in *C. keiskei* from leaf, flower, and root tissue

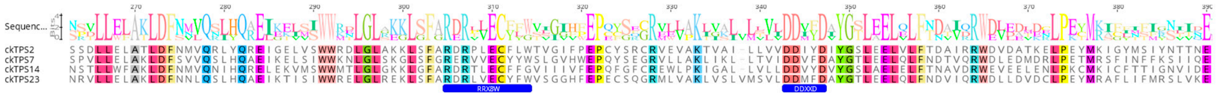

Supplementary Fig S8. The top four ckTPS genes and their conserved domain of RRX8W and DDXXD domains are aligned sequences

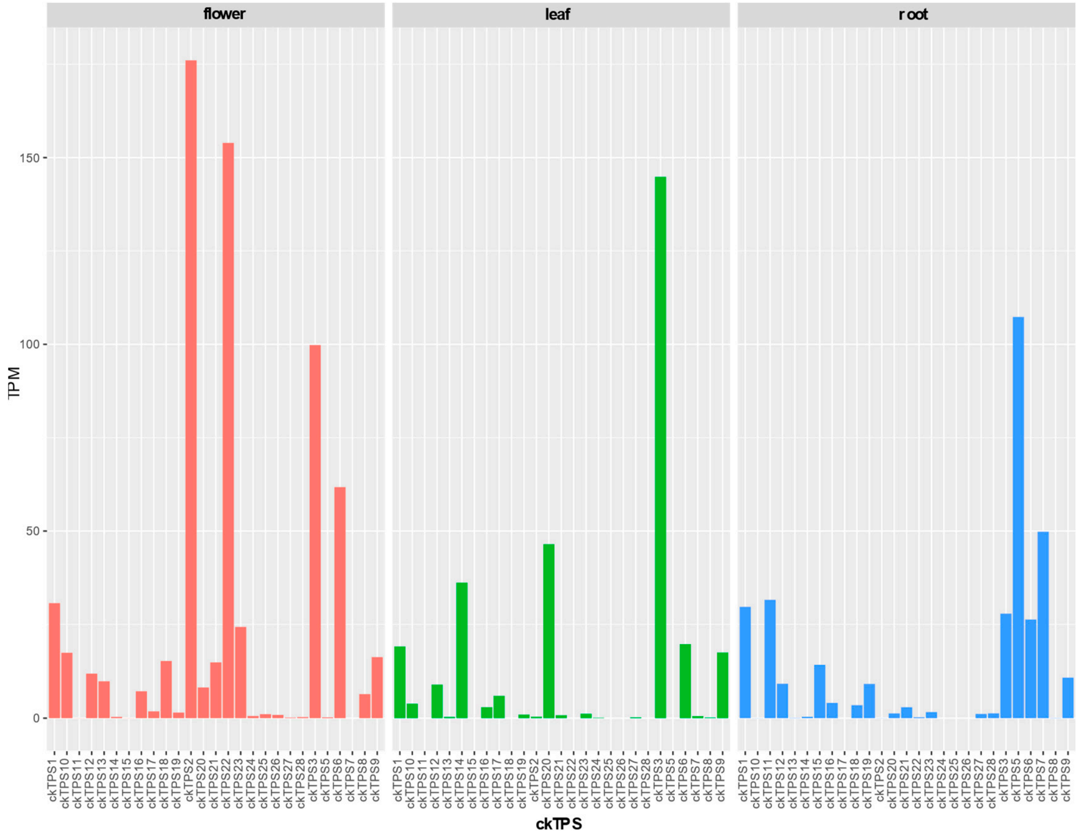

Supplementary Fig S9. Bar plots of TPM value of RSEM assessment to the ckTPS genes align, and mapping of *Convallaria keiskei* reads quantification analysis.

Supplementary Table S2: List of highly expressed genes in the flower, leaf and root of *C. keiskei* and their role in the plants

Transcript ID gene name	Enzyme name	Metabolite name	Highly expression	Function

ckTPS2	Geraniol synthase	Monoterpene	Flower	Floral scent, defense mechanism, stress response, medicinal properties
ckTPS23	Germacrene (Ger D) synthase	sesquiterpene	Flower	Floral scent, defense mechanism, stress response, medicinal properties
ckTPS13	Nerolidol synthase	Nerolidol (sesquiterpene)	Flower	Floral scent, defense mechanism, stress response, medicinal properties
ckTPS7	Valencene synthase	Valencene (sesquiterpene)	Flower and Root	Floral scent, citrus aroma, insect repellent, stress response, medicinal properties
ckTPS4, 5, 10	Trans-ocimene	Monoterpene	Flower and Root	Floral scent, plant communications, insect attraction and repellence, stress response
ckTPS16, 27, 28	Kaurene synthase	Diterpenoid	Flower, Leaf and Root	Precursor for plant hormone, gibberellins (GAS); plant growth and developmental process
ckTPS11	Trans-ocimene	Monoterpene	Leaf and Root	Floral scent, plant communications, insect attraction and repellence, stress response
ckTPS6, 14, 17, and 18	linalool synthase	Monoterpene	Flower	Floral scent, defense mechanism, stress response, medicinal properties

Supplementary Table S4. Top upregulated in leaf, flower, and root of *C. keiskei* using DEGs analysis

unigenes	leaf	flower	root	description
leaf				
TRINITY_DN865_c1_g2	6.405638032	-1.26017827	-5.14545977	plastocyanin 1
TRINITY_DN5007_c0_g1	6.062331346	-2.22416415	-3.8381672	Aldolase superfamily protein
TRINITY_DN1598_c0_g2	5.989057744	-1.61804756	-4.37101018	galactinol synthase 4
TRINITY_DN21_c0_g1	5.787968911	-1.11100312	-4.67696579	sedoheptulose-bisphosphatase
TRINITY_DN1598_c0_g1	5.758316198	-2.11187826	-3.64643794	galactinol synthase 4
TRINITY_DN286_c0_g1	5.589524419	-1.40296613	-4.18655829	phosphoribulokinase
TRINITY_DN6998_c0_g1	5.236912939	0.337748707	-5.57466165	glyceraldehyde-3-phosphate dehydrogenase B subunit
TRINITY_DN5666_c0_g1	5.217190977	-1.43364094	-3.78355004	carbonic anhydrase 1
TRINITY_DN576_c0_g2	5.123788974	-0.615654	-4.50813498	Chalcone-flavanone isomerase

TRINITY_DN4758_c0_g1	5.087092031	-1.23966453	-3.8474275	hydroxypyruvate reductase
**flower**				
TRINITY_DN6014_c0_g1	-5.56887964	5.709013887	-0.14013425	lipid transfer protein 3
TRINITY_DN849_c1_g1	-2.18822494	4.806813116	-2.61858817	terpene synthase (ckTPS2)
TRINITY_DN2242_c0_g1	-0.97237219	4.599409052	-3.62703686	lipid transfer protein 3
TRINITY_DN1432_c0_g1	-1.10304837	4.14912112	-3.04607275	GDSL-like Lipase/Acylhydrolase
TRINITY_DN62992_c0_g1	-1.96567148	4.127189522	-2.16151804	glycosylphosphatidylinositol-anchored lipid protein transfer 1
TRINITY_DN9736_c0_g1	-1.8117389	4.033430645	-2.22169174	xyloglucan hydrolase 24
TRINITY_DN23_c0_g6	-3.47605855	3.93988504	-0.46382649	geranylgeranyl reductase
TRINITY_DN2504_c0_g1	-0.77319421	3.812773352	-3.03957915	expansin A8
TRINITY_DN1646_c0_g1	-2.69359083	3.676653456	-0.98306263	lipid transfer protein 6
TRINITY_DN2326_c0_g1	-1.23223387	3.393354818	-2.16112095	auxin response factor 8
**root**				
TRINITY_DN16033_c0_g1	-4.80268602	-2.23076249	7.033448508	Curculin-like lectin protein
TRINITY_DN95_c1_g1	-4.52951627	-1.91574356	6.445259824	magnesium transporter 4
TRINITY_DN16762_c1_g3	-4.18808015	-1.99380172	6.181881871	Lipase/lipooxygenase2C
TRINITY_DN11363_c0_g1	-4.48233334	-0.1852887	4.667622039	O-methyltransferase protein
TRINITY_DN2596_c0_g1	-0.39342283	-3.73521135	4.128634174	Glycosyl hydrolases 32 protein
TRINITY_DN7764_c0_g1	-1.71046321	-2.34420809	4.054671301	allene oxide synthase
TRINITY_DN5605_c1_g1	-0.69811789	-3.2526453	3.95076319	metallothionein 2A
TRINITY_DN38071_c0_g2	-2.11958887	-1.72672195	3.846310825	methylesterase PCR A
TRINITY_DN4058_c0_g1	-1.4682735	-2.09961065	3.567884145	terpene synthase (ckTPS7)
TRINITY_DN4084_c0_g1	-1.54330777	-1.94182255	3.485130324	ferulic acid 5-hydroxylase 1

plants

Supplementary Table S5. Top down-regulated genes of leaf, flower, and root of *C. keiskei* using DEGs analysis

unigenes	leaf	flower	root	description
**leaf**				
TRINITY_DN6014_c0_g1	-5.56887964	5.709013887	-0.14013425	lipid transfer protein 3
TRINITY_DN16033_c0_g1	-4.80268602	-2.23076249	7.033448508	Curculin-like protein
TRINITY_DN95_c1_g1	-4.52951627	-1.91574356	6.445259824	magnesium transporter 4
TRINITY_DN11363_c0_g1	-4.48233334	-0.1852887	4.667622039	O-methyltransferase family protein
TRINITY_DN16762_c1_g3	-4.18808015	-1.99380172	6.181881871	Lipase/lipooxygenase2C PLAT/LH2 protein
TRINITY_DN976_c0_g3	-3.80086743	0.80665996	2.99420747	lipoxygenase 1
TRINITY_DN2025_c0_g1	-3.57718121	3.357453233	0.219727975	Plantinvertase/pectin methylesterase inhibitor
TRINITY_DN23_c0_g6	-3.47605855	3.93988504	-0.46382649	geranylgeranyl reductase
**flower**				
TRINITY_DN10_c0_g1	3.303408162	-4.68488032	1.381472157	O-methyltransferase family protein
TRINITY_DN4250_c0_g1	0.822584011	-4.04768682	3.225102806	O-methyltransferase family protein
TRINITY_DN2596_c0_g1	-0.39342283	-3.73521135	4.128634174	Glycosyl hydrolases family 32 protein
TRINITY_DN395_c0_g1	3.577352098	-3.68923201	0.111879912	O-methyltransferase family protein
TRINITY_DN6674_c0_g1	1.532701824	-3.36812021	1.835418385	beta glucosidase 16
TRINITY_DN5605_c1_g1	-0.69811789	-3.2526453	3.95076319	metallothionein 2A
TRINITY_DN1598_c1_g1	0.792285086	-3.21725581	2.424970728	galactinol synthase 1
TRINITY_DN7468_c0_g1	-0.43588815	-2.89105522	3.326943376	glutathione S-transferase TAU 20
TRINITY_DN3105_c0_g1	1.846122569	-2.86769847	1.0215759	Glycosyl hydrolases family 32 protein
**root**				
TRINITY_DN6674_c0_g2	4.525353113	1.442954261	-5.96830737	beta glucosidase 17
TRINITY_DN6998_c0_g1	5.236912939	0.337748707	-5.57466165	glyceraldehyde-3-phosphate dehydrogenase B subunit
TRINITY_DN865_c1_g2	6.405638032	-1.26017827	-5.14545977	plastocyanin 1
TRINITY_DN3898_c1_g1	4.989146557	-0.12373722	-4.86540933	glyceraldehyde 3-P dehydrogenase A
TRINITY_DN21_c0_g1	5.787968911	-1.11100312	-4.67696579	sedoheptulose-bisphosphatase
TRINITY_DN6873_c0_g1	2.71974644	1.929143933	-4.64889037	Glutathione S-transferase protein
TRINITY_DN499_c0_g1	4.051126712	0.47087128	-4.52199799	Glucose-6-phosphate/phosphate translocator-like protein
TRINITY_DN576_c0_g2	5.123788974	-0.615654	-4.50813498	Chalcone-flavanone isomerase protein
TRINITY_DN1598_c0_g2	5.989058	-1.61805	-4.37101	galactinol synthase 4
